# Understanding the Barriers to Pediatric Oncologist Engagement and Accrual to Clinical Trials in National Cancer Institute–Designated Community Oncology Research Programs

**DOI:** 10.1200/JOP.19.00707

**Published:** 2020-05-12

**Authors:** David S. Dickens, Michael E. Roth, Brad H. Pollock, Anne-Marie Langevin

**Affiliations:** ^1^ Department of Pediatrics, Division of Hematology, Oncology and Bone Marrow Transplantation, University of Iowa, Holden Comprehensive Cancer Center, Iowa City, IA; ^2^ Department of Pediatrics Patient Care, Division of Pediatrics, The University of Texas MD Anderson Cancer Center, Houston, TX; ^3^ Department of Public Health Sciences, School of Medicine, University of California, Davis, Comprehensive Cancer Center, Davis, CA; ^4^ Department of Pediatrics, Division of Hematology/Oncology, The University of Texas Health San Antonio, San Antonio, TX

## Abstract

**PURPOSE:**

Clinical trial participation leads to progress in cancer care. Principal investigators (PIs) and clinical research associates (CRAs) play key roles in the provision and maintenance of clinical trial portfolios at their sites. Previous studies have evaluated the educational and resource needs of adult oncology providers, but nothing to date has focused on providers of pediatric oncology care. We aimed to identify the educational needs and clinical trial participation barriers at National Cancer Institute Community Oncology Research Program (NCORP) Children’s Oncology Group (COG) sites to improve the quality of site investigator engagement.

**METHODS:**

Quality improvement surveys of pediatric clinical research staff at NCORP sites were performed. The first was a web-based inquiry of NCORP COG PIs and lead CRAs to assess their general understanding of NCORP organizational structure and needs. The second survey of COG PIs was conducted by one-on-one telephone interviews aimed at identifying specific barriers to physician engagement and patient enrollment in clinical trial research.

**RESULTS:**

The majority of NCORP COG PIs and CRAs (63%) reported an incomplete understanding of NCORP structure, with approximately half expressing interest in developing stronger collaborations and engagement. Most NCORP COG PIs reported at least one shared barrier to clinical trial enrollment (78%), with inadequate protected time and research support (39% each) being the most frequently cited barriers.

**CONCLUSIONS:**

Contributions to pediatric cancer clinical research at COG NCORP sites could be enhanced through improved education, resources, and time allocation.

## INTRODUCTION

Clinical trial enrollment benefits the individual patient by offering a chance for improved survival while also providing benefit to future patients by defining optimal treatments.^1-6^ Although the majority of surveyed US oncologists and patients with cancer view clinical trial enrollment as beneficial,^7,8^ most US patients with cancer do not participate in clinical trials. Efforts to explain this paradox have pointed to a variety of organizational, patient, and physician barriers to clinical trial enrollment.^9^ One organizational reality recognized decades ago was that the majority of US patients with cancer were not treated at academic institutions and simply did not have access to clinical trials.^10^ In response to this access disparity, the National Cancer Institute (NCI) funded a series of community-based oncology research programs throughout the United States to transport state-of-the-art cancer research into community settings that serve a large and diverse patient population.^11^ Initiated in 1983 as the Community Clinical Oncology Programs (CCOPs), adding the Minority-Based CCOPs in 1990 and being re-organized as the NCI Community Oncology Research Program (NCORP) in 2014, these programs aim to address oncology needs at community, minority/underserved, and rural minority/underserved sites. While NCORPs have shown substantial success with regard to providing clinical trial access to the greater US cancer population, the challenges of clinical trial enrollment have been similar to that seen in academia.^12-14^

One patient subgroup recently subject to much-needed scrutiny has been the adolescent and young adult (AYA) population, given the relative stagnation in outcome improvement for many of the malignancies that afflict this age-group and the association of age with poor clinical trial participation.^15^ Within the AYA population, clinical trial availability and enrollment differ by organizational factors such as community versus academic sites and adult versus pediatric programs.^3-5,16-18^ Many NCORPs possess pediatric and adult components with shared infrastructure while offering academic institution–like clinical trial availability. It was previously hypothesized that NCORPs provide the ideal organizational design to optimize AYA enrollment. Unfortunately, when assessed for Children’s Oncology Group (COG) studies, there is evidence that CCOP-funded community sites enroll proportionally fewer AYA patients compared with non-CCOP sites.^19^ Emerging data suggest similar enrollment results when non-COG studies are considered.^19a^ Taken together, these results raised concerns about the level of engagement, integration, and recognition of pediatric and AYA components within their respective NCORPs.

The COG NCORP Committee (CNC) represents NCORP site interests within COG’s administrative and scientific activities. The CNC monitors and develops mechanisms to ensure robust treatment trial accrual from NCORP sites with an added focus on the development and conduct of studies in cancer prevention and control, cancer health disparities, and cancer care delivery research. The findings reported in this article were generated from quality improvement projects aimed at gathering phenomenological information from COG NCORP pediatric oncology clinical research staff on barriers to clinical trial engagement.

## METHODS

### Study Design

The CNC conducted two quality improvement surveys during the first NCORP grant cycle between December 2016 and January 2018. The first survey was conducted electronically through SurveyMonkey (SurveyMonkey, San Mateo, CA) between December 2016 and January 2017 and targeted the NCORP COG principal investigators (PIs) and lead clinical research associates (CRAs) at all 38 COG pediatric oncology programs. The second survey of NCORP COG site PIs was conducted through telephone interview led by D.S.D. and A.-M.L. between October 2017 and February 2018. All NCORP COG site PIs received e-mail notification from the interviewers. Nonresponders received a second e-mail notification. Responders then arranged for a time to conduct the telephone survey with the interviewer. Descriptive statistics are reported. These activities were reviewed by The University of Texas Health San Antonio institutional review board and deemed as quality improvement projects because they were designed to implement processes that will improve patient care at NCORP COG programs.

### Instrumentation

Survey questions were codeveloped by CNC leadership and believed to be relevant to the understanding of the needs of clinical research investigators (Table 1). The first web-based survey consisted of 10 questions designed to assess a general understanding of NCORP structure and the CNC’s role in helping responders to achieve their accrual goals. The second 7-question telephone survey focused on barriers to clinical trial enrollment and collected PI experiences, observations, and ideas both in general and in specific domains. Responders were not required to answer every question and could make more than one comment for barrier assessment questions.

**TABLE 1.**
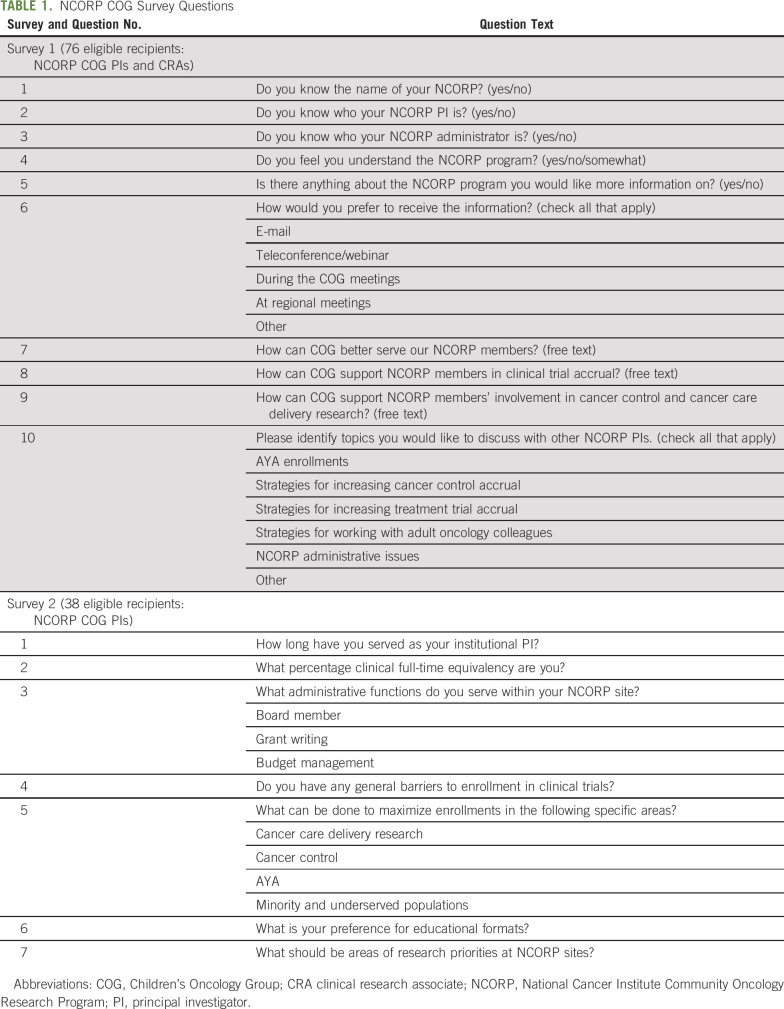
NCORP COG Survey Questions

## RESULTS

### Survey 1

Of the 76 individuals surveyed, 44 responded, for a participation rate of 58%. The majority of NCORP COG PIs and CRAs (63%) did not understand or somewhat understood the NCORP in general. Portions were unfamiliar with key aspects of their NCORP, including the names of their NCORP PI (16%), the NCORP administrator (30%), and the NCORP itself (23%). Responders reported the need to receive education on strategies to work with medical oncologists (55%) and to increase accrual for cancer control studies (55%), AYA patients (48%), and therapeutic trials (38%). E-mails (80%), in-person sessions during the COG meeting (62%), and teleconferences/webinars (52%) were cited as the preferred modalities for receiving information. Other suggestions for program support were more timely information about the general functioning and funding of NCORPs, increasing access to multilanguage-translated consents, and increasing the number of active therapeutic and cancer control trials.

### Survey 2

Overall, 23 of 38 NCORP COG PIs agreed to participate in a phone interview (61% response rate). The median number of years participants served as PI was 5 (30% held the PI position for ≤ 2 years), and they reported limited informational handoffs from previous institutional PIs. The mean reported clinical effort was 80%. With regard to NCORP board structure, budget, or grant writing, 8 (35%) of 23 NCORP COG PIs were not involved with any activity, 5 (22%) were engaged with all, and the rest had partial involvement. General barriers to enrollment were identified by 18 (78%) of the 23 responding NCORP COG PIs. The two most frequently cited barriers were insufficient research assistance and insufficient protected time to effectively manage patient recruitment efforts and the research portfolio (9 of 23; 39%). Other cited barriers were internally competitive studies, institutional research offices, consent fatigue for both investigators and patients, electronic health records, insurance coverage, and lack of patient and family knowledge about clinical trials. Prominent trends within the pre-identified specific research domains included the benefit of AYA program coordinators, the advantage of culturally matched in-person translators, an inability to prioritize cancer control studies, and the lack of understanding of cancer care delivery research (Table 2).

**TABLE 2.**
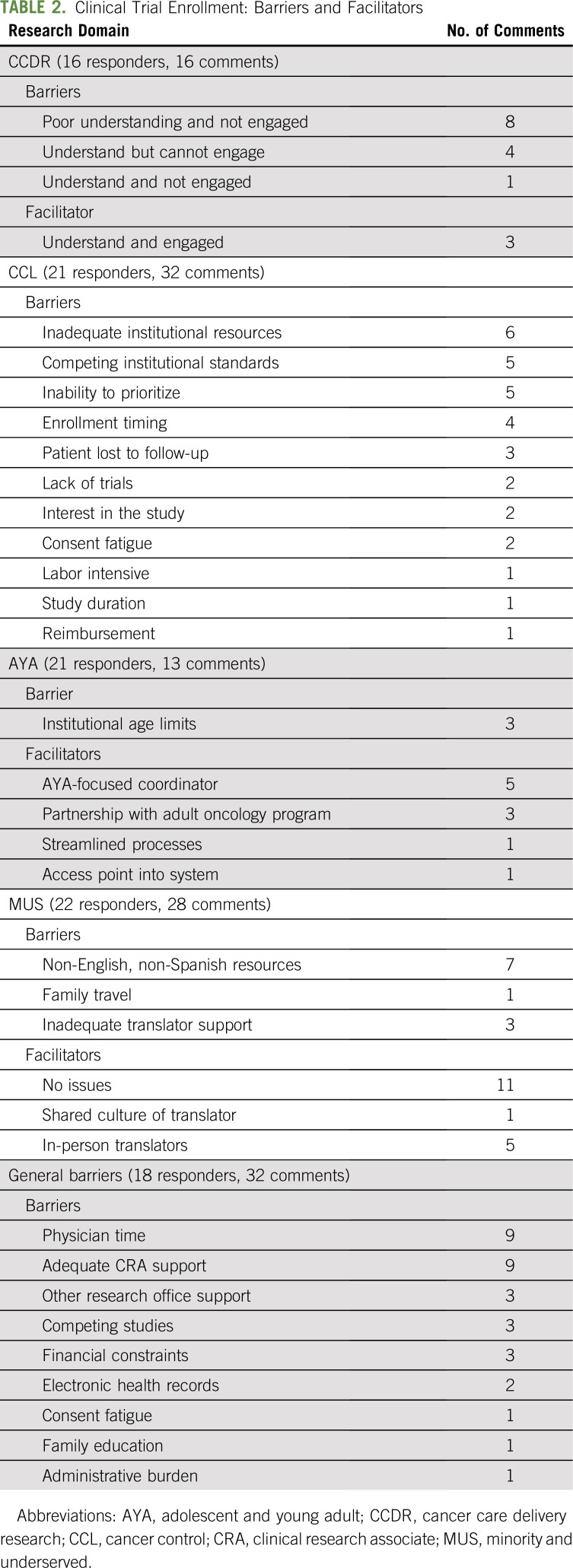
Clinical Trial Enrollment: Barriers and Facilitators

## DISCUSSION

Given the positive association between clinical trial enrollment and survival, efforts to eliminate organizational, patient, and physician barriers are essential to maximizing outcomes for patients. In 2015, Freyer and Seibel^20^ introduced a conceptual model entitled Clinical Trial Pathway to Enrollment to highlight the steps required for clinical trial enrollment.^21^ Within every step of the process (a trial must first exist and then become available, presented, and accepted), oncologists serve an important facilitative role. Therefore, understanding physician attitude, attributes, and barriers are critical to improving clinical trial enrollment. Providers for adult patients with cancer have reported clinical trial awareness, rigid protocol design, insufficient staffing, beneficence toward patient care, and issues with time prioritization as barriers to clinical trial enrollment.^7-9,13,21^ Our findings suggest that NCORP pediatric oncologists experience similar limitations that fall into the areas of education, time, and resource allocation as the most prominent issues.

In response, the CNC is creating a series of Accrual Enhancement Activities to help pediatric NCORP investigators and their institutions to become vibrant components and contributors to the NCORP’s operation and mission, that is, strong accrual of minority/underserved patients and strong contributors to symptom management and cancer care delivery research. The framework includes a tripartite approach of enhancing education, engagement with leadership, and infrastructure support. First, suboptimal PI education is being addressed through more systematic exportation of transferable success strategies. Peer-to-peer mentoring, webinars, and embedded sessions at national meetings are in varying stages of development to address these deficiencies in knowledge. Second, COG PI incorporation into their respective NCORP leadership structure has been valued by those who practice in such a setting and will be nurtured by the CNC. Recently conducted cancer care delivery research launch calls between the NCI and NCORP PIs and administrators revealed that NCORP administrations were often unaware of COG activities (B. H. Pollock, personal communication, July 2019). To that effect, the CNC is currently developing a newsletter that will be distributed to COG PIs, lead CRAs, and their respective NCORP administrators and PIs. It is believed that through integrative dialogue between NCORP site leadership and administration that embedded COG PIs can better manage, prioritize, and advocate for their program needs. Third, the CNC strongly supports NCI initiatives to address the most frequently self-reported barrier to physician engagement—time—by providing funding for NCORP site leadership involvement in supporting the clinical research enterprise.

Our findings suggest that additional support is needed to enhance physician engagement and activity in clinical trial enrollment. The challenges of providing institutional and professional investments are ongoing and contextual to the complex environmental, financial, and professional realities of limited resources and increasing health care provider responsibilities.^18^ Limitations inherent to this project’s design include potential sampling bias given survey response rates of 58% and 61%; nonresponders may have different experiences and opinions. In addition, our findings were limited to NCORP COG providers who as a group represent approximately 20% of all COG sites and do not include medical oncologists. Finally, because these surveys were developed to improve program quality, they lack vigorous validation and are specific to the survey sample, so the findings may have limited generalizability, particularly beyond the domain of NCORP COG members.

Despite these limitations, we believe that identifying education, time, and resources as barriers to clinical research engagement is credible because these findings closely match medical oncology reports and are consistent with ongoing phenomena such as CRA turnover, site case reimbursements, and the challenges of clinical research effort valuation (D. S. Dickens, personal communication, September 2019). We hope that it is the response to our findings, a provision of accrual enhancement activities, that will not only maximize the benefits experienced by patients at COG NCORP sites but also pertain to adult and pediatric, community, and academic oncologists alike.
